# A comprehensive phenotypic and genotypic evaluation of Spanish groundnuts from diverse crosses to identify superior and stable donors for fresh seed dormancy

**DOI:** 10.1038/s41598-024-64681-6

**Published:** 2024-07-01

**Authors:** Kirti Rani, K. Gangadhara, B. C. Ajay, Praveen Kona, Narendra Kumar, S. K. Bera

**Affiliations:** 1 ICAR-Directorate of Groundnut Research, Junagadh, Gujarat India; 2 ICAR-National Bureau of Plant Genetic Resources, Regional Station, Jodhpur, Rajasthan India; 3 ICAR-Central Tobacco Research Institute, Research Station Kandukur, Kandukur, Andhra Pradesh India

**Keywords:** Genetics, Molecular biology, Climate sciences

## Abstract

Breeding high yielding groundnut cultivars with 2–3 weeks of fresh seed dormancy, particularly in Spanish-type cultivars, enhances the sustainability of agriculture in groundnuts. In this context, we conducted a comprehensive phenotypic and genotypic evaluation of advanced breeding lines developed in the genetic background of Spanish types. By employing multi-phenotyping and marker data, we identified PBS 15044, 16004, 16013, 16015, 16016, 16017, 16020, 16021, 16026, 16031, 16035, 16037, 16038, 16039, 16041, and 16042 with 2–3 weeks dormancy (> 90%).The various parametric and non-parametric estimates identified the stable fresh dormant genotypes with one or more superior economic trait. PBS 16021, 15044, 16038, and 16039 identified with high hundred pod weight (HPW) were also reported having high intensity of dormancy (> 90% for up to 3 weeks); PBS 15044, 16016, PBS 16038 and PBS 16039 with high hundred kernel weight (HKW) also reported with up to 3 weeks fresh seed dormancy; and PBS 16013, 16031, and 16038 with up to 3 weeks fresh seed dormancy had high shelling percentage (SP). They can be used to develop lines with the desired level of dormancy, and high yields, by designing appropriate breeding strategies.

## Introduction

Groundnuts, scientifically known as *Arachis hypogaea* L., are widely grown oilseed crops that belong to the Fabaceae or Leguminosae family. They are cultivated in more than 100 countries across the globe. Groundnuts have become popular because of their versatility and diverse applications in human nutrition. They are used in the production of cooking oil, confectionery items, dietary food, and livestock feed. Based on the branching pattern, flower arrangement, kernel, and pod attributes, cultivated groundnuts have been categorized into three major market types, i.e., Virginia (subsp. *hypogaea* var. *hypogaea*), Spanish (subsp. *fastigiata* var. *vulgaris*), and Valencia (subsp. *fastigiata* var. *fastigiata*)^1,2^. In general, Virginia genotypes have a late maturation period and their seeds have different lengths of dormancy. On the other hand, Spanish and Valencia types are early maturing and their seeds do not have dormancy^3^. Spanish cultivars, widely grown in the semi-arid regions of Asia and Africa, account for 60% of global groundnut production. Nevertheless, premature rainfall prior to harvest can cause in situ germination (pre-harvest sprouting) of seeds, leading to a decrease in yield by 10–20% and perhaps up to 50% in Spanish varieties. These losses have a negative impact on the quality of the kernels, market pricing, and increase the vulnerability to pathogen infection and aflatoxin contamination ^4,5^. An effective strategy for addressing this issue is enhancing high yielding groundnut variety by introducing a period of 2–3 weeks of seed dormancy. This would enable the plants to withstand the negative impact of rain during the period between maturity and harvest^4,6^. So, fresh dormant groundnuts provide significant advantages to farmers by allowing them to delay crop harvesting in the event of unexpected rains, therefore minimizing losses due to pre-harvest sprouting (PHS). So, to develop high yielding Spanish bunch cultivars with 2–3 weeks of fresh seed dormancy (FSD) is an important objective for plant breeders. But, any phenotypes are the manifestations of Genotype (G), Environment (E), and their interactions. The environmental conditions such as humidity, soil texture and fertility, precipitation, and temperature may all play a role in the yield fluctuation and variable phenotypic expressions ^7,8^. Performance of genotypes varies with different environmental conditions. This interaction between genotype and season (GSI) which is responsible for yield instability or variation in phenotypic expression has been reported in several crops^9^. Significant season-to-season variations in fresh seed dormancy have been observed due to genotypic differences, and GSI is most common because of the differential expression of genotypes across seasons, which may complicate the selection process of a genotype for a target trait. In such cases, stability analysis provides an excellent solution for the relative performance of genotypes across seasons. Plant breeders commonly apply multi-season trials (METs) to examine the relative performance of genotypes across seasons. So, for newly generated fresh dormant advance lines, a prerequisite is to undertake a multi-season trial to determine the superior and stable groundnut genotypes*.* Accordingly, the objective of the present investigation is to identify fresh dormant genotypes that exhibit superior yield performance that is consistent and high across diverse environmental. To investigate genotypic stability for intensity of dormancy (IOD) for up to 3 weeks and considerable season-to-season yield fluctuations use of stability models such as Additive main effect and multiplicative interaction (AMMI), AMMI based stability index (ASI), BLUP based predicted means, and estimation of yield relative to the environment maximum (YREM) of advanced breeding lines (ABLs) were evaluated. This study intended to identify potential ABL(s) that might consistently perform for the targeted traits throughout the crop seasons.

## Materials and methods

### Plant materials and experimental site

A comprehensive evaluation was conducted on 29 advanced breeding lines (ABLs) of groundnut developed from diverse crosses followed by selection at advanced stage (Table S1) along with three released dormant check varieties viz., Girnar 3, Dh 86 and TPG 41 at ICAR-Directorate of Groundnut Research (DGR), Junagadh, Gujarat, India in a medium black calcareous (12% CaCO_3_), clayey, Ustochrept soil. Test materials were sourced from our own gene bank at ICAR-Directorate of Groundnut Research, Junagadh. The test materials used in the present study are numbered and used with same nomenclature throughout the manuscript. All the plant material was obtained and developed at ICAR-DGR, Junagadh and no specific permissions are required as they are our own advanced breeding lines developed from crossing at ICAR-DGR. The experiment was arranged in a randomized complete block design (RCBD) with three replications. Each accession was planted in a single row of 3 m in length, with a spacing of 60 cm between rows × 10 cm between plants. Geographically, the experimental site is located at 21^o^31' N latitude, 70^o^36' N longitude at an altitude of 63 m above mean sea level comprising black calcareous soil. Proper crop management practices, such as the application of a recommended dose of fertilizers (50–40–25 kg NPK/ha) were complied.

### Phenotyping protocols and parameter estimated for fresh seed dormancy

**Figure 1:**
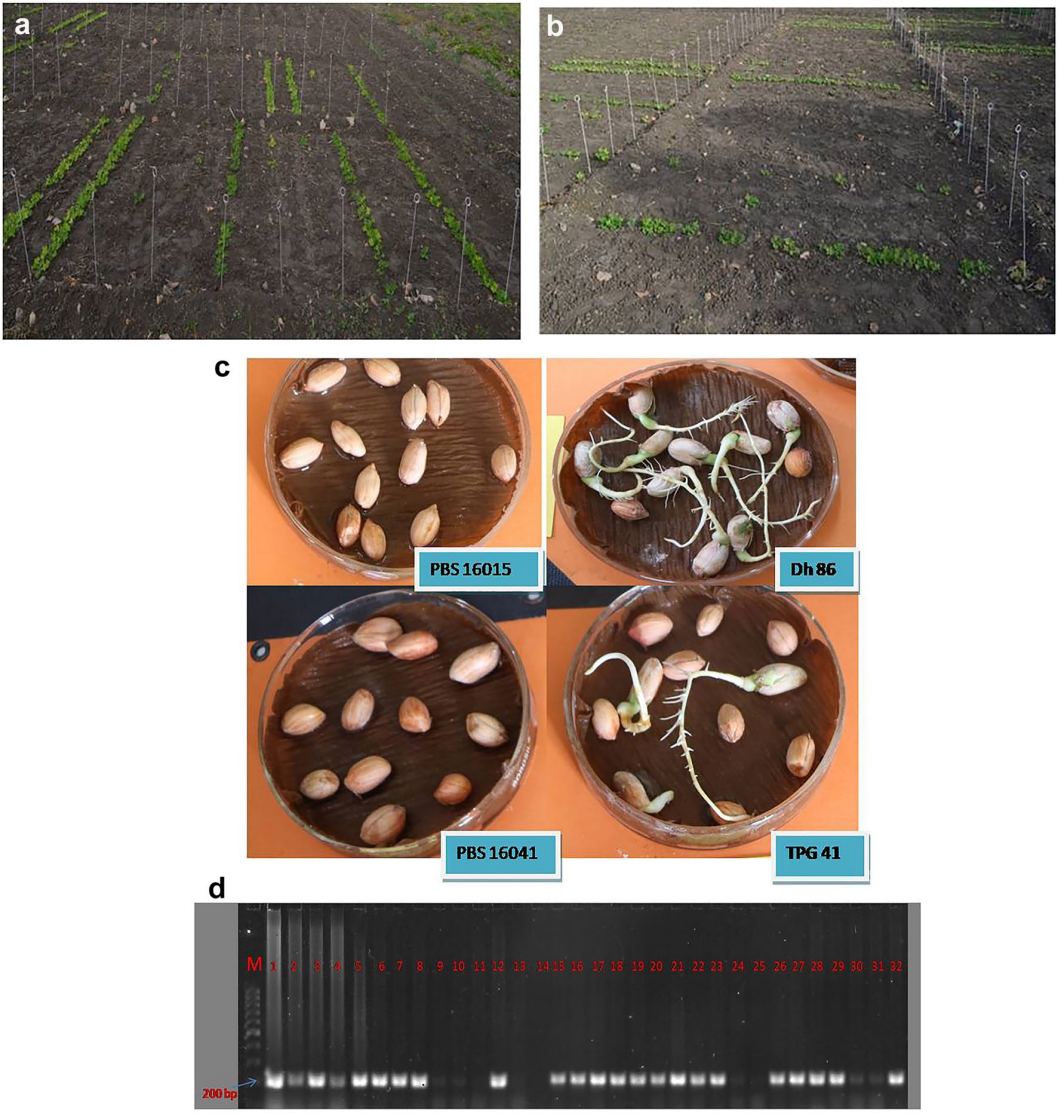
Phenotyping variation for fresh seed dormancy (**a,b**) Field germination tests of fresh and mature kernels from freshly harvested pods. (**c**) Laboratory germination tests in petriplate. (**d**) Advanced Breeding Lines validated on a validation panel comprising a marker GMFSD-1, an allele specific marker for fresh seed dormancy^10^.

The genotypes were evaluated for fresh seed dormancy for three seasons viz*., Kharif* 2021(kh21), summer-2022 (sum22) and *Kharif* 2022 (kh22), by testing them under (i) field testing, (ii) laboratory conditions and (iii) genotyping using allele specific marker associated with fresh seed dormancy, the fresh dormant ABLs were genotyped on a validation panel comprising a GMFSD-1*,* an allele specific marker for fresh seed dormancy as described by Kumar et al. 2019^10^. For *Kharif* (kh) experiment, the seeding process occurred during the first two weeks of July, while the harvesting activities were conducted during the first two weeks of November. Whilst, for summer (sum) experiment, the seeding process occurred during the first two weeks of February, and the harvesting activities were conducted during the first week of June. For, (i) field test, seeds from freshly harvested pods of groundnut were treated with carbendazim (3 g/kg of seed) and sown in randomized complete block design with three replications. Each genotype was planted in a single row of 3 m in length, with a spacing of 60 cm between rows and 10 cm between plants (Fig. 1a, b). For, (ii) laboratory screening, standard operating procedure (SOP) for germination test was followed as proposed by Janila et al. (2018)(Fig. 1c)^11^.The freshly harvested seeds from each genotype were treated with fungicide, treated seeds were placed in petriplate and watered regularly. The data on number of seeds germinated were recorded at weekly interval for up to 21 days to calculate intensity of dormancy (IOD) as described by Gangadhara et al. 2022 (intensity of dormancy 15 days after sowing in the field (IOD15F), 21 days after sowing in the field (IOD21F), 15 days after sowing in petriplate (IOD15L), 21 days after sowing in petriplate (IOD21L)^8^. For field germination tests moisture was maintained in the soil at field capacity throughout the experiment. The genotypes for germination tests were harvested at maturity as indicated by blackening of inner parenchyma of the pod^12^. To study fresh seed dormancy, a sample of mature pods were randomly selected and shelled immediately after harvesting and precaution was taken to prevent any damage of the testa, cotyledons and embryo while removing seeds from pods. Freshly harvested mature seeds were used for phenotyping. The mean pod moisture content of summer harvested produce was 38% and *Kharif* season harvested pods had 32% moisture content.

### Evaluation of fresh dormant advanced breeding lines (ABLs) for yield and its components

The set of 29 Spanish bunch advanced breeding lines of groundnut that were screened for FSD were also evaluated for yield (pod yield per plant; PYLP) and its component traits (direct and positively contributing traits with yield) such as hundred pod weight (HPW), hundred kernel weight (HKW) and shelling percentage (SP) in grams for three seasons namely, *Kharif* 2020, *Kharif* 2021, and *summer* 2022. Standard agricultural practices and plant protection measures were adopted for healthy crop production. The data were recorded from five randomly selected plants of each genotype under each replication.

### Statistical analysis

The data analysis was performed using version 4.2.1 of the R statistical software^13^. The data on intensity of dormancy (IOD), yield and yield-related variables were analysed using a combined analysis of variance (ANOVA) to evaluate the existence of genotype by season interaction (GSI). All the data underwent log transformation for normalisation prior to analysis. In order to facilitate AMMI biplot modelling, each year and location was considered as a distinct and autonomous environment and were analysed using packages "agricolae".^.^ Homogeneity of error mean squares as revealed by Levene’s (Levene, 1960) test (p = 0.85) provided the statistical validity to pool the data from all seasons to perform AMMI analysis^14^. AMMI analysis was undertaken to detect and characterize genotype × season interaction (GSI)^15^. The signal rich component of GSI (GSI_Signal_) sum of squares was computed as GSISS noise-GSI_Noise_, where GSI_noise_ = GSI degrees of freedom × error mean squares from the AMMI ANOVA^16^.

### Estimation of yield relative to the environmental maximum (YREM) of genotypes

The mean value relative to the environmental (season in our study) maximum (YREM)^17^. was computed as Y_ij_ = X_ij_/MAX_j_, where Y_ij_ and X_ij_ are the YREM and mean value, respectively, of genotype ‘i’ and season ‘j’. MAX_j_ is the maximum value for any ‘genotype’ observed in season _j_. YREM was estimated using MS excel software. YREM is a special type of standardized estimate of genotype’s performance with nullified year main effect. We performed one way ANOVA based on BLUP and YREM estimates to estimate the significance/otherwise of differences among fresh dormant advanced breeding lines.

### Estimation of statistics to assess stability of genotypes differing in performance

The AMMI-based stability parameters (ASTABs), such as ASI were computed^18^. The formula was derived from an AMMI Stability Index (ASI), as originally proposed by Jambhulkar et al. 2014^18^. Although AMMI can be considered the best tools for simultaneously visualizing the mean grain yield and genotype stability, these cannot provide the exact numerical information required for comparison. Therefore, biplots alone cannot be relied on where more than two PCs are required to interpret a considerable proportion of GSI. The stability parameters in this study, namely, AMMI stability index (ASI) utilize all significant PCs for their estimation and were also considered for calculating simultaneous selection index (SSI).

Simultaneous stability index (SSI) incorporate mean yield and stability index in a single equation and is calculated as: SSI = rASI + rY where, rASI is the rank of ASI value and rY is the rank of mean yield of genotype across environments. The AMMI and stability indices were determined using R statistical software, version 3.4.1^13^^.^ The genotypes with lower and higher average ASI- and SSI- based ranks were interpreted to exhibit high and low performing genotypes, respectively.

### Handling plant materials and methods

The collection and handling of plant and methods were in accordance with all the relevant guidelines.

## Results

### Intensity of dormancy

#### Mean performance and ANOVA

A comprehensive analysis was performed in order to dissect the main effects and assess the interrelationships within and between the factors of variation. The study revealed significant genotypic differences for IOD (15F, 21F, 15L and 21L) within each season (Table S2). The present study also revealed the existence of significant genotype × season interaction differences for the IOD tested under different germination tests. Consequently, higher estimates of broad-sense heritability were also observed for IOD in each season that depicts the higher proportion of phenotypic variation contributed due to genetic values. The genotypic accuracy of selection (As), which measures the correlation between predicted and observed values were recorded from 0.98 to 1.00. In addition coefficient of variation (CV) ranged from 5.61 to 18.21 (Table S2).The mean values of IOD15 in the field ranged from 72.36 (sum22) to 81.09 (kh21); IOD21F ranged from 67.81 (kh22) to 73.49 (kh21); IOD15L ranged from 69.86 (sum22) to 77.92 (kh22) and IOD 21L ranged from 65.59 (sum22) to 66.77 (kh22) (Table S3). The intensity of dormancy ranged from 8.24% (PBS 16028) to 100% (PBS 16015, 16021, 16041, and 16045) for IOD15F; 12.96% (PBS 16044) to 100% (PBS 16004, 16015, 16016, 16031, 16037, and 16041) for IOD15L; 0% (PBS 16028) to 100% (PBS 16015, 16041) for IOD21F; and 2.59% (PBS 16032) to 100% (PBS 16015, 16016 and 16041) for IOD21L (Table S3). In the present study, the results showed that around 17 breeding lines had more than 90% mean intensity of dormancy from both field and laboratory germination tests for 2 weeks. Among these, 16 breeding lines had cumulative IOD more than 90% for upto 3 weeks after sowing.

### The validation of fresh seed dormant groundnuts with allele-specific markers associated with fresh seed dormancy

The validation of fresh dormant genotypes via marker-assisted screening in addition to phenotypic screening was also performed in this study to identify accessions with 2–3 weeks of dormancy. So, these 29 ABLs phenotyped for germination percentage were also genotyped with allele specific marker GMFSD1 to validate the dormancy. GMFSD1 is an allele specific marker (B05_111598196) developed from the chromosome B05, through QTL-Seq analysis^10^. In the present study, this marker showed clear polymorphism between dormant (> 80% IOD, 21 DAS) and non-dormant parents (< 10% IOD, 21DAS) and co-segregated with the dormant phenotype equivalent or more than 80 percent intensity of dormancy (Fig. 1d). The marker exhibited remarkable efficiency in distinguishing between nondormant and dormant genotypes of Spanish types. However, there were a few exceptions wherein the marker data were not in accordance with the phenotyping data. In the validation panel, except genotypes, PBS 16020, 16021, 16024, 16026, 16027, 16042, 16044, 16052, and 16053 all other ABLs along with checks showed positive band with marker GMFSD1. Among these, only genotypes, PBS 16027, 16044, and 16052 were non-dormant (< 20% IOD at 3 weeks after sowing) in germination tests under field and laboratory conditions. Other genotypes though dormant in phenotyping tests showed negative results in genotyping panel. Further, PBS 16025, 16028, 16029, and 16032, though reported non-dormant (< 20% IOD at 3 weeks after sowing) from phenotyping test showed positive amplification in validation panel.

### AMMI-stability analysis for fresh seed dormancy

The mean genotypic values from three different seasons were subjected to AMMI ANOVA analysis. AMMI ANOVA indicates a larger contribution of sum of squares (SS) attributable to additive genotype main effects for fresh seed dormancy (88.36% to 94.46%) (Table 1). Further, mean squares (MS) attributable to main effects of genotypes (GEN), seasons (ENV) and multiplicative GSI effects (ENV: GEN) were significant for IOD (except non-significant environment effects for IOD21L). The variance caused by GSI was further subdivided into variance caused by signal and noise (Table 2). Partitioning GSI into signal and noise portions indicated that the signal portion was higher (54.39–93.27%) than the noise portion (6.73–45.61%) for FSD. Hence, AMMI analysis is appropriate for data sets where-in SS due to GSI_Signal_ are at least as large as those due to additive genotype main effects^16^.

**Table 1:** Additive Main effects and Multiplicative Interaction (AMMI) analysis.

	df	IOD15F		IOD21F		IOD15L		IOD21L	
		MSS	% SS	MSS	% SS	MSS	% SS	MSS	% SS
ENV	2	1868.30**	1.19	948.30**	0.48	1558.70**	0.93	56.60	0.02
REP(ENV)	6	83.80	0.16	73.20	0.11	187.40	0.34	184.10	0.24
GEN	31	8931.10**	88.36	11,748.50**	91.71	9633.30**	89.54	13,860.30**	94.46
ENV:GEN	62	432.50**	8.56	359.90**	5.62	305.60**	5.68	163.30**	2.23
PC1	32	788.36**	94.10	652.33**	93.60	401.76**	67.90	191.50**	60.5
PC2	30	52.88**	5.90	47.95	6.40	203.00**	32.10	133.31*	39.5
Residuals	186	29.10	1.73	44.40	2.08	62.90	3.51	74.5	3.05

		PYLP		HPW		HKW		SP	
ENV	2	487.43**	46.00	7173.00**	19.38	798.75**	9.02	5036.4**	35.45
REP(ENV)	6	1.25	0.36	190.20	1.54	61.89	2.10	91.7	1.94
GEN	31	14.59**	21.34	1320.30**	55.29	305.29**	53.41	352.3**	38.44
ENV:GEN	62	5.75**	16.81	145.20**	12.16	41.41**	14.49	52.00**	11.34
PC1	32	9.73**	87.40	217.79**	77.40	54.70**	68.20	83.01**	82.40
PC2	30	1.50	12.60	67.86	22.60	27.24	31.80	18.85	17.60
Residuals	186	1.76	15.49	46.3	11.63	20	20.99	19.6	12.83

*df* degrees of freedom, *IOD15F* intensity of dormancy 15 days after sowing (field), *IOD21F* intensity of dormancy 21 days after sowing (field), *IOD15L* intensity of dormancy 15 days after sowing (laboratory), *IOD21L* intensity of dormancy 21 days after sowing, laboratory, *MSS* mean sum of squares, *% SS* percentage sum of squares, *% Var* contribution towards total variation, *PYLP* pod yield/plant, *HPW* hundred pod weight, *HKW* hundred kernel weight, *SP* shelling percentage.

*,**Significant at 1% and 5% respectively.

**Table 2:** Estimates of sum of squares due to signals and noises by using AMMI model in groundnut.

	IOD15F		IOD21F		IOD15L		IOD21L	
	SS	% Var	SS	% Var	SS	% Var	SS	% Var
GSIsignal	25,009.80	93.27	19,560.20	87.66	15,046.20	79.42	5508.00	54.39
GSInoise	1804.20	6.73	2752.80	12.34	3899.80	20.58	4619.00	45.61

	PYLP		HPW		HKW		SP	
GSIsignal	247.12	69.37	6134.40	68.12	1327.50	51.70	2006.60	62.28
GSInoise	109.12	30.63	2870.60	31.88	1240.00	48.30	1215.20	37.72

GSI, genotype × season interaction.

### AMMI biplot analysis

**Figure 2:**
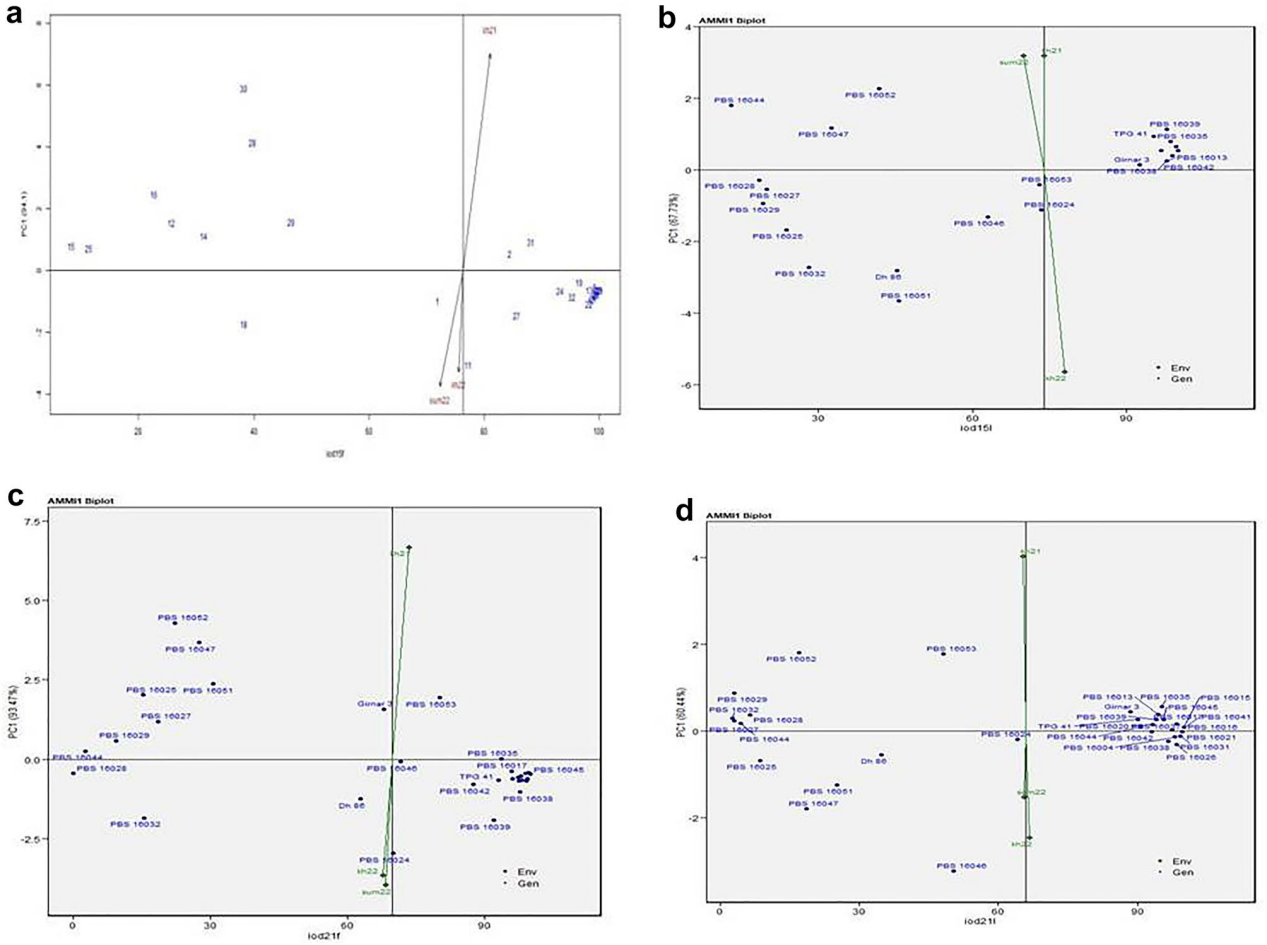
AMMI1 biplot trait *vs* principal component 1 (PC1) for (**a**) IOD15F, (**b**) IOD15L, (**c**) IOD21F, (**d**) IOD21L of 32 groundnut genotypes evaluated under three seasons viz., kh21, sum22, and kh22.

The AMMI stability showing the relationship between experimental genotypes and test environments for fresh seed dormancy (IOD15F, IOD15L, IOD21F and IOD21L) across three seasons is presented in Fig. 2a–d. The season kh21 was farthest from biplot origin, with long vectors representing strong interaction forces, while kh22 and sum22 were nearer to the origin and had shorter vectors with weak interaction forces for IOD15F, IOD21L, IOD21L, while for IOD15L, kh22 with long vector represented strong interaction forces^19^. The biplot depicted that for IOD15F and IOD15L entries, TPG 41, PBS 15044, 16004, 16013, 16015, 16016, 16017, 16020, 16021, 16026, 16031, 16035, 16037, 16038, 16039, 16041, 16042 and 16045 had more values; while, PBS 16025, PBS 16027, 16028, 16029, and 16044 had less values. From cumulative results of field and laboratory, for IOD 21 days after sowing, PBS 16004, 16015, 16016, 16017, 16021, 16026, 16031, 16035, 16037, 16038, 16039, 16041, 16042, and 16045 and were superior in performance. Out of these genotypes, most of genotypes especially PBS16035 for IOD15F; PBS 16038 and PBS 16042 for IOD 15L; PBS 16017, 16035 for IOD21F; PBS 16016, 16020, 16021, 16031, 16037 and 16042; for IOD21L was placed on zero PC1 scoreline, or closer to PC1 line, making it more stable with wider adaptation to the test environments. For IOD15F, IOD15L, IOD21F and IOD21L first PC1 explained 94.1%, 67.73%, 93.74%, 60.44% respectively, of genotype x environment interaction.

### Comparative performance and stability of genotypes differing in intensity of dormancy as assessed by AMMI based stability index

The visualization of AMMI1 biplots was difficult since 32 genotypes were studied with many of them overlapping, creating a fuzzy figure, so AMMI stability index was computed. The variety with the lowest AMMI stability Index (ASI) calculated from the IPCA axis and IPCA scores is the most stable^20,21^. Based on mean performance for IOD tested 15 days after sowing cumulative in field and laboratory conditions, best genotypes were PBS 16004, 16015, 16021, 16037 and 16041. However, for IOD15F, based on ASI, PBS 16017, 16021, 16026, 16035, and Girnar 3 were stable and PBS 15044, 16013, 16026, 16028, 16038 and 16042 for IOD tested in the laboratory germination tests. PBS 16013, 16015, 16016, 16021 and 16041 for IOD21F; PBS 16015, 16016, 16021, 16031, and 16041 for IOD21L were superior in mean performance. For stability, as per ASI rankings, PBS 16015, 16016, 16017, 16035, 16044 for IOD21F and PBS 16015, 16016, 16021, 16041 and 16042 for IOD21L were stable across seasons (Table 3). Since, ASI give an idea about only the stability of genotypes and does not provide any information about mean performance so, simultaneous selection index (SSI) was computed^22^ by adding the ranks of stability parameter and mean performance. The least SSI is considered as most stable with high mean values, whereas high SSI is considered as least stable with low means^22^. Based, on cumulative results of field and laboratory conditions, for IOD 15 days after sowing, PBS 16015, 16021, 16041 and 16045 were most desirable based on SSI. While, for IOD 21 days after sowing based on cumulative results, PBS 16015, 16016, and 16041 was most desirable (Table 3).

**Table 3:** Comparative ranks of AMMI stability index (ASI) and simultaneous selection index (SSI) for intensity of dormancy in groundnut.

S. no.	Genotypes	IOD 15F			IOD 21F			IOD 15L			IOD 21L		
		Rasi	rY	SSI	rASI	rY	SSI	rASI	rY	SSI	rASI	rY	SSI
1.	Dh 86	21	23	44	23	23	46	30	24	54	24	23	47
2.	Girnar 3	3	21	24	24	22	46	1	19	20	18	19	37
3.	PBS 15044	12	11	23	10	8	18	8	16.5	24.5	7	17	24
4.	PBS 16004	11	5	16	16	7	23	15	4	19	9	8	17
5.	PBS 16013	14	6.5	20.5	18	5	23	2	14.5	16.5	17	13	30
6.	PBS 16015	6	2.5	8.5	7	1.5	8.5	12	4	16	4	2	6
7.	PBS 16016	9	8	17	6	6	12	14	4	18	5	2	7
8.	PBS 16017	2	9	11	4	14	18	17	8	25	10	6.5	16.5
9.	PBS 16020	17	10	27	15	13	28	16	16.5	32.5	19	15	34
10.	PBS 16021	5	2.5	7.5	13	4	17	11	4	15	2	4	6
11.	PBS 16024	30	22	52	30	21	51	24	20	44	22	20	42
12.	PBS 16025	26	29	55	28	29	57	26	28	54	27	27	54
13.	PBS 16026	4	14	18	14	12	26	7	9	16	13	6.5	19.5
14.	PBS 16027	24	28	52	22	27	49	18	29	47	11	30.5	41.5
15.	PBS 16028	16	32	48	8	32	40	6	31	37	20	28	48
16.	PBS 16029	29	30	59	12	30	42	22	30	52	25	30.5	55.5
17.	PBS 16031	22	13	35	19	10	29	9	4	13	6	5	11
18.	PBS 16032	28	26	54	25	28	53	29	27	56	14	32	46
19.	PBS 16035	1	16	17	1	15	16	20	11	31	26	12	38
20.	PBS 16037	15	6.5	21.5	11	11	22	13	4	17	12	9	21
21.	PBS 16038	19	12	31	21	9	30	4	12.5	16.5	8	10	18
22.	PBS 16039	23	15	38	26	17	43	23	14.5	37.5	23	14	37
23.	PBS 16041	7	2.5	9.5	9	1.5	10.5	10	4	14	3	2	5
24.	PBS 16042	10	18	28	20	18	38	3	12.5	15.5	1	16	17
25.	PBS 16044	13	31	44	3	31	34	27	32	59	21	29	50
26.	PBS 16045	8	2.5	10.5	5	3	8	5	10	15	15	11	26
27.	PBS 16046	25	20	45	2	20	22	28	22	50	32	21	53
28.	PBS 16047	31	25	56	31	25	56	25	26	51	28	25	53
29.	PBS 16051	27	24	51	29	24	53	32	23	55	31	24	55
30.	PBS 16052	32	27	59	32	26	58	31	25	56	29	26	55
31.	PBS 16053	20	19	39	27	19	46	19	21	40	30	22	52
32.	TPG 41	18	17	35	17	16	33	21	18	39	16	18	34

*IOD15F* intensity of dormancy 15 days after sowing (field), *IOD21F* intensity of dormancy 21 days after sowing (field), *IOD15L* intensity of dormancy 15 days after sowing (laboratory), *IOD21L* intensity of dormancy 21 days after sowing, laboratory, *rY* ranks based on mean performance, *rASI* ranks based on AMMI stability index, *SSI* simultaneous selection index.

### Identification of stable and productive genotypes by BLUP

**Figure 3:**
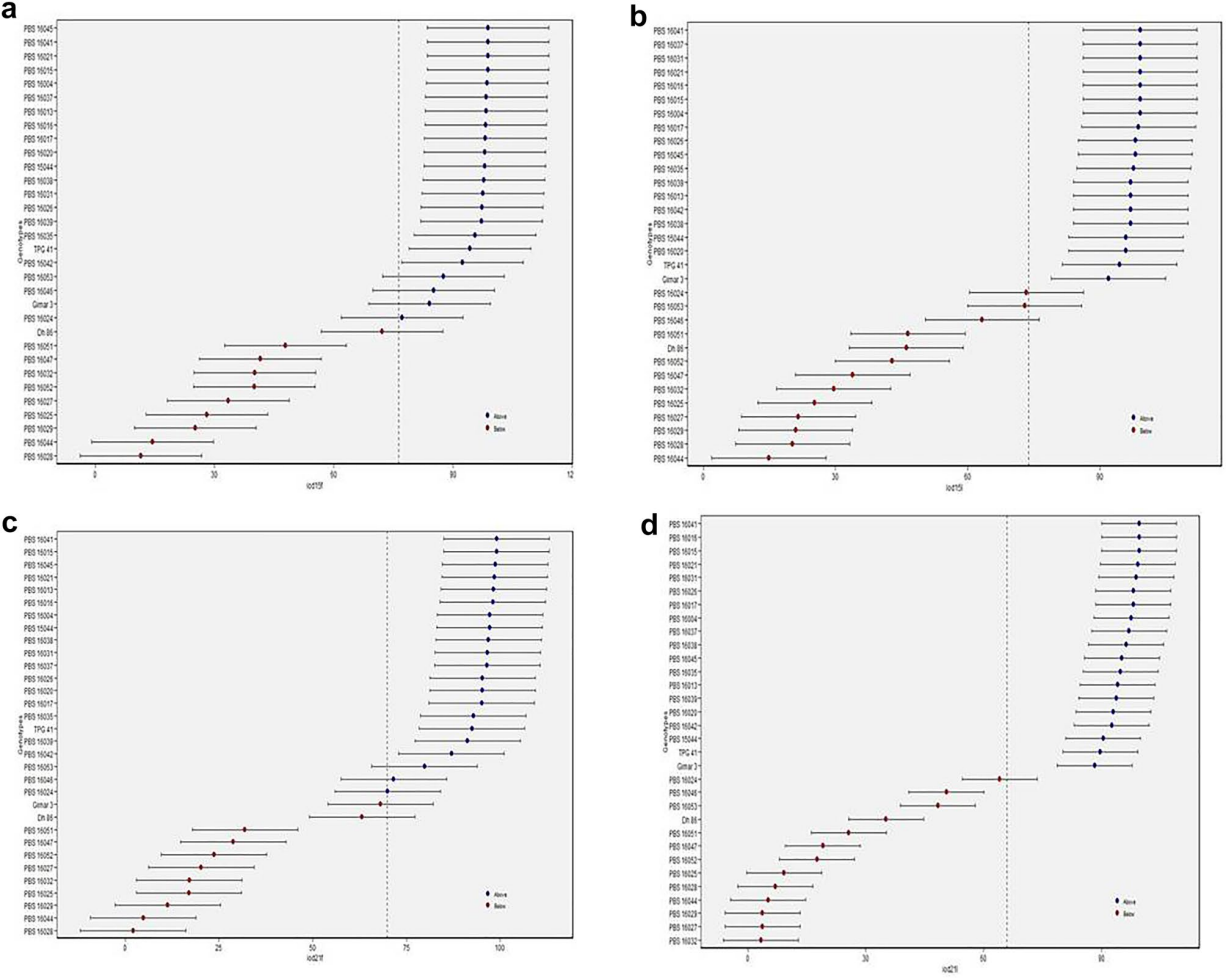
Identification of stable and productive genotypes by BLUP of 32 groundnut genotypes evaluated under three seasons viz., kh21, sum22, and kh22 (**a**) IOD15F, (**b**) IOD15L, (**c**) IOD21F, (**d**) IOD21L.

The presence of a significant G × S interaction suggests that one genotype's yield may be superior to another genotype in one environment but inferior in another, implying that BLUP analysis is necessary^23^. From BLUP estimates, PBS 15044, 16004, 16013, 16015, 16016, 16017, 16020, 16021, 16026, 16031, 16035, 16037, 16038, 16039, 16041, 16042, 16045, TPG 41 and Girnar 3 had the highest predicted means above grand mean among the tested genotypes for IOD15DAS with no considerable differences among them. The same set of genotypes also had the highest predicted means among the tested genotypes for IOD 21DAS (Fig. 3a–d).

### Comparative performance of genotypes differing in intensity of dormancy as assessed by YREM

YREM is an indicative of magnitude of crossover GSI. Therefore, in the absence of crossover GSI, the average YREM of a genotype tested across seasons (*Kharif* and summer) must be 1.0. Any departure of YREM of a genotype from 1.0 is attributable to loss in its attainable mean performance due to crossover GSI^17^. For example, if a genotype has an average of 0.90 across-seasons YREM, then 10% of its attainable performance is lost due to crossover GSI. In our study, PBS 16004, 16015, 16021, and 16041 with an average YREM of 1.0 were highly stable with zero cross over interactions and PBS 16016, 16017, 16020, 16026, 16031, 16037, 16038, 16039, with an average YREM value of 0.98–0.99 were stable for intensity of dormancy 15 days after sowing. Similarly, PBS 16015, and 16041 with YREM of 1.0 were highly stable; and PBS 16004, 16016, 16017, 16021, and 16031 were comparatively stable more intensity of dormancy 21 days after sowing based on cumulative results of field and laboratory germination tests (Table 4).

**Table 4:** Estimates of YREM’s of groundnut genotypes for intensity of dormancy.

	IOD 15F				IOD 21F				IOD 15L				IOD 21L			
	kh20	kh21	sum22	Avg	kh20	kh21	sum22	Avg	kh20	kh21	sum22	Avg	kh20	kh21	sum22	Avg
Dh 86	0.69	0.76	0.71	0.72	0.57	0.68	0.64	0.63	0.33	0.70	0.32	0.45	0.31	0.43	0.30	0.35
Girnar 3	0.93	0.88	0.72	0.84	0.84	0.64	0.56	0.68	0.94	0.96	0.88	0.93	0.91	0.87	0.88	0.89
PBS 15044	0.98	1.00	0.99	0.99	0.98	0.98	0.99	0.98	0.98	0.97	0.96	0.97	0.91	0.92	0.89	0.91
PBS 16004	0.99	1.00	1.00	1.00	0.97	0.98	1.00	0.98	1.00	1.00	1.00	1.00	0.96	0.98	1.00	0.98
PBS 16013	0.98	1.00	1.00	0.99	0.98	1.00	1.00	0.99	1.00	1.00	0.93	0.98	0.97	0.93	0.93	0.94
PBS 16015	1.00	1.00	1.00	1.00	1.00	1.00	1.00	1.00	1.00	1.00	1.00	1.00	1.00	1.00	1.00	1.00
PBS 16016	0.99	1.00	0.99	0.99	0.99	0.99	0.99	0.99	1.00	1.00	1.00	1.00	1.00	1.00	1.00	1.00
PBS 16017	1.00	0.99	0.99	0.99	0.97	0.94	0.97	0.96	1.00	0.99	1.00	1.00	0.99	0.97	1.00	0.99
PBS 16020	0.98	1.00	1.00	0.99	0.95	0.93	1.00	0.96	0.97	0.97	0.97	0.97	0.93	0.90	0.97	0.93
PBS 16021	1.00	1.00	1.00	1.00	0.98	1.00	1.00	0.99	1.00	1.00	1.00	1.00	0.99	1.00	1.00	1.00
PBS 16024	0.58	0.87	0.87	0.77	0.50	0.81	0.79	0.70	0.63	0.86	0.71	0.73	0.61	0.60	0.71	0.64
PBS 16025	0.43	0.21	0.13	0.26	0.35	0.07	0.04	0.15	0.20	0.40	0.11	0.24	0.04	0.21	0.00	0.09
PBS 16026	0.98	0.99	0.98	0.98	0.95	0.96	0.98	0.96	0.97	1.00	1.00	0.99	0.96	1.00	1.00	0.99
PBS 16027	0.45	0.23	0.26	0.31	0.32	0.14	0.10	0.19	0.24	0.28	0.08	0.20	0.04	0.04	0.00	0.03
PBS 16028	0.19	0.06	0.00	0.08	0.00	0.00	0.00	0.00	0.21	0.24	0.10	0.19	0.09	0.08	0.02	0.06
PBS 16029	0.47	0.14	0.07	0.23	0.18	0.11	0.00	0.10	0.22	0.30	0.06	0.19	0.09	0.00	0.00	0.03
PBS 16031	0.96	1.00	1.00	0.99	0.96	0.97	1.00	0.98	1.00	1.00	1.00	1.00	0.98	1.00	1.00	0.99
PBS 16032	0.29	0.56	0.30	0.38	0.04	0.24	0.18	0.15	0.26	0.52	0.07	0.28	0.04	0.03	0.00	0.03
PBS 16035	0.98	0.97	0.94	0.96	0.98	0.92	0.91	0.94	0.99	0.97	1.00	0.99	0.98	0.88	1.00	0.95
PBS 16037	0.98	1.00	1.00	0.99	0.97	0.96	1.00	0.97	1.00	1.00	1.00	1.00	0.97	0.96	1.00	0.97
PBS 16038	0.97	1.00	1.00	0.99	0.93	1.00	1.00	0.98	0.97	1.00	0.97	0.98	0.94	0.99	0.97	0.97
PBS 16039	0.95	1.00	1.00	0.98	0.80	0.96	1.00	0.92	1.00	0.93	1.00	0.98	0.94	0.88	1.00	0.94
PBS 16041	1.00	1.00	1.00	1.00	1.00	1.00	1.00	1.00	1.00	1.00	1.00	1.00	1.00	1.00	1.00	1.00
PBS 16042	0.93	0.96	0.91	0.93	0.85	0.88	0.90	0.88	0.97	1.00	0.97	0.98	0.92	0.93	0.93	0.93
PBS 16044	0.22	0.11	0.01	0.11	0.08	0.00	0.00	0.03	0.21	0.03	0.14	0.13	0.04	0.00	0.09	0.04
PBS 16045	1.00	1.00	1.00	1.00	1.00	0.99	1.00	1.00	1.00	1.00	0.97	0.99	0.97	0.93	0.97	0.96
PBS 16046	0.79	0.89	0.89	0.86	0.75	0.60	0.80	0.72	0.42	0.77	0.70	0.63	0.26	0.63	0.62	0.50
PBS 16047	0.77	0.22	0.20	0.40	0.60	0.11	0.11	0.28	0.31	0.28	0.39	0.33	0.04	0.26	0.26	0.19
PBS 16051	0.64	0.37	0.39	0.46	0.53	0.14	0.24	0.31	0.37	0.77	0.23	0.46	0.18	0.46	0.12	0.25
PBS 16052	0.89	0.16	0.10	0.38	0.60	0.03	0.03	0.22	0.70	0.29	0.27	0.42	0.30	0.11	0.10	0.17
PBS 16053	1.00	0.89	0.76	0.88	0.99	0.74	0.67	0.80	0.79	0.80	0.60	0.73	0.62	0.50	0.32	0.48
TPG 41	0.93	0.97	0.96	0.95	0.92	0.92	0.96	0.93	0.99	0.92	0.94	0.95	0.91	0.88	0.91	0.90

*IOD15F* intensity of dormancy 15 days after sowing (field), *IOD21F* intensity of dormancy 21 days after sowing (field), *IOD15L* intensity of dormancy 15 days after sowing (laboratory), *IOD21L* intensity of dormancy 21 days after sowing, laboratory.

### Mean *vs* stability for fresh seed dormancy

**Figure 4:**
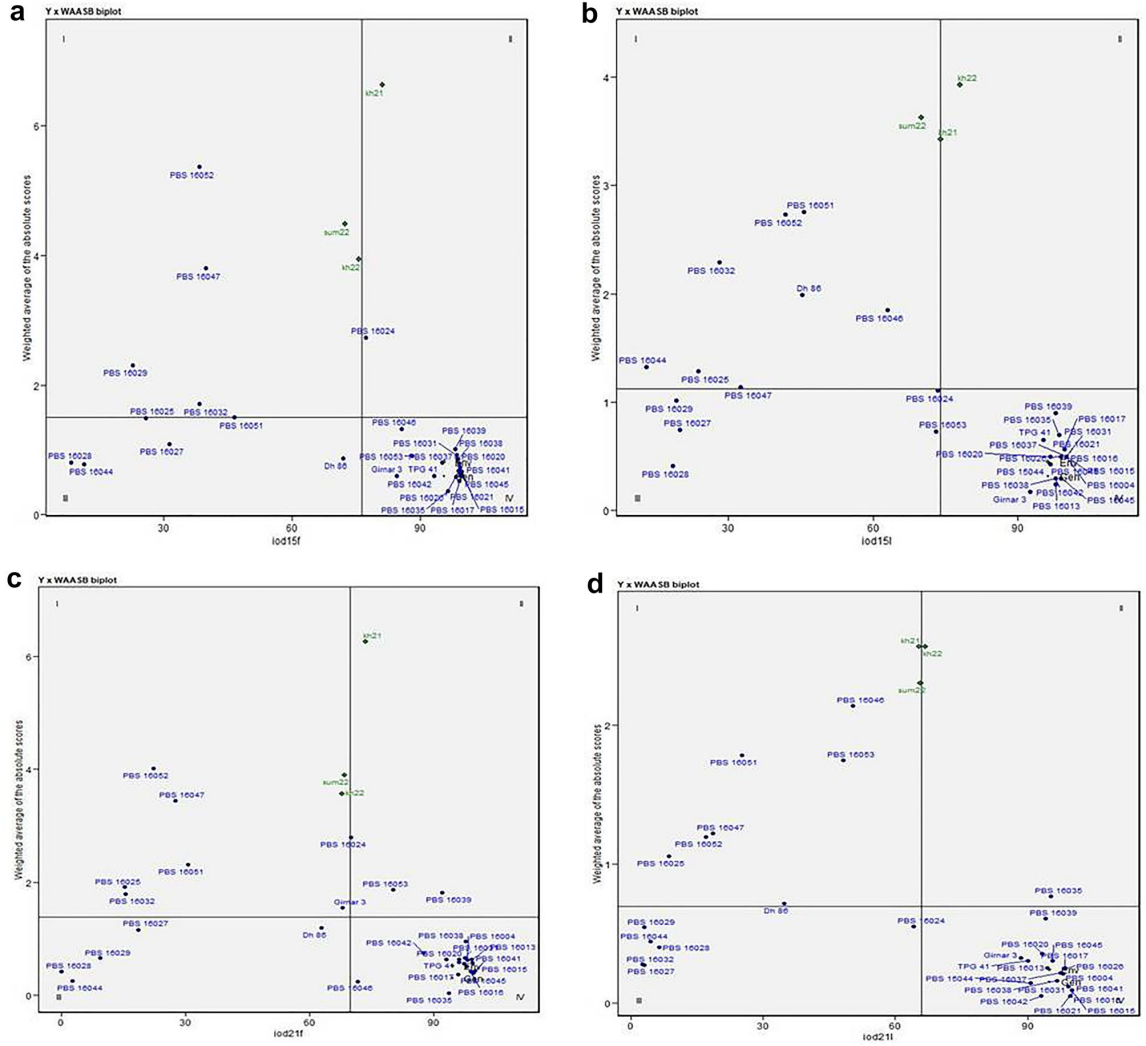
Biplots for (**a**) IOD15F, (**b**) IOD15L, (**c**) IOD21F, (**d**) IOD21L *vs.* weighted average absolute scores for the best linear unbiased predictions of 32 groundnut genotypes evaluated across the seasons.

A joint interpretation of individual trait performance and stability of genotypes across the seasons is presented in the four quadrants of Y × WAASB biplot in Fig. 4a–d. The genotypes or environments (seasons) placed in quadrant I are unstable or environments with high discrimination ability and low productivity below the grand mean. In quadrant II, the productivity of the genotype is above the grand mean but unstable. The environments in quadrant II were good discriminating environments with high magnitudes of the response variable. Genotypes in quadrant III have low productivity but stable due to the lower values of WAASB. The environments in quadrant III is considered poorly productive and with low discrimination ability. The genotypes in quadrant IV are highly productive and broadly adapted due to the high magnitude of the response variable and high stability performance. The present study includes the seasons, sum22 and kh22 in quadrant I, followed by kh21 in quadrant II of the WAASB biplot for fresh seed dormancy. This indicates that all three seasons were good discriminating environments for fresh seed dormancy. The season sum22 is unfavorable and less productive with the high discriminating ability for the studied trait and is placed in quadrant I. PBS 16024 (IOD15F); PBS 16039 and 16053 (IOD21F); PBS 16035 (IOD21L) are genotypes places in quadrant II, indicating high mean but variable performance. These genotypes displayed superior performance in either of the seasons, but not all. Genotypes in quadrant III have low productivity but stable due to the lower values of WAASB such as, PBS 16027, 16028, 16044 and Dh 86 (IOD15F); PBS 16024, 16027, 16028, 16029, and 16053 (IOD15L); PBS 16027, 16028, 16029, 16044 and Dh 86 (IOD21F); and PBS 16024, 16027, 16028, 16029, 16032, and 16044 (IOD21L). The genotypes placed in quadrant I are unstable and have low productivity below the grand mean viz*.,* PBS 16029, 16032, 16047, and 16052 (IOD15F); PBS 16025, 16032, 16044, 16046, 16051, 16052 (IOD15L); PBS 16025, 16032, 16047, 16051, and 16052 (IOD21F); and PBS 16025, 16046, 16047, 16051, 16052, and 16053 (IOD21L). All the remaining genotypes are included in quadrant IV of WAASB biplot. These genotypes are highly stable, but at the cost of their comparatively poor performance. This group is relevant in means that these genotypes had consistently superior trait values across seasons.

### Yield and related attributes

#### AMMI-stability analysis for yield and related traits

The PYLP, ranged from 4.24 (PBS 16017) to 9.15 g (PBS 16028); 54.77 g (PBS 16026) to 96.90 (PBS 16032) for HPW; 27.70 g (PBS 16051) to 49.84 (PBS 16032) for HKW; and 42.29 (PBS 16026) to 68.04% (PBS 16038) for SP. The top five best genotypes were PBS 16028, 16032, 16046, 16053, and 16025 for PYLP; PBS 16032, 16021, 16039, 15044, and 16038 for HPW; PBS 16032, 16039, 16038, 16016, and 15044 for HKW; PBS 16038, 16032, Dh 86, 16013, and 16031 for SP. AMMI ANOVA indicates a relatively higher contribution of sum of squares (SS) attributable to additive genotype main effects, 55.29% for HPW, 53.41% for HKW, and 38.44% for SP (Table 1). In contrast for PYLP, analysis observed the greater contribution of environments (46%) followed by genotypic effects (21.34%) and 16.81% of the total variation was explained through interaction effects. The variance caused by GEI was further subdivided into variance caused by signal and noise. Consequently, for yield and related attributes signal portion was higher (51.70–69.37%) than the noise portion (30.63–48.30%) (Table 2). Further, the significant contribution of genotypes and GSI_Signal_ to total variation among the genotypes across seasons, for yield and related traits, justify the application of AMMI and BLUP models to our data to understand patterns of GSI. The multiplicative component of AMMI models consists of the singular value/multiplication factor of IPCA, the genotype eigenvector, and the environment eigenvector^24^. In the present study, the first PCs explained 68% (HKW) to 87% (PYLP) of GSI, indicating that most variation was captured by the first component, whereas second component accounted for 12.60 to 31.80 percent of the GSI, indicating a significant contribution of environment on genotype and trait expression performance.

#### AMMI1 biplot analysis for yield and attributes

**Figure 5:**
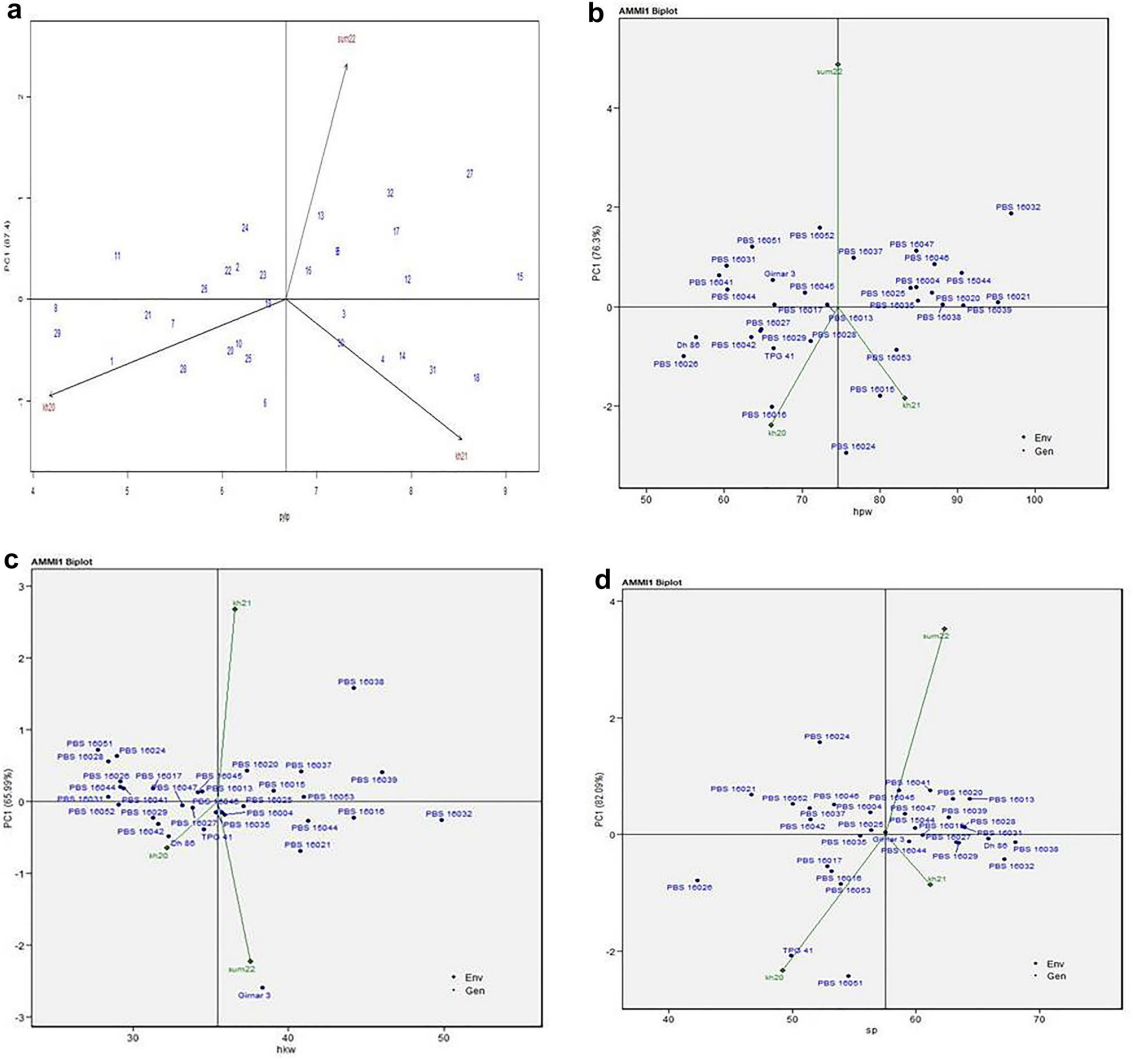
AMMI1 biplot trait vs. principal component 1 (PC1) for (**a**) PYLP, (**b**) HPW, (**c**) HKW and (**d**) SP, of 32 groundnut genotypes evaluated under three seasons viz., kh21, sum22, and kh22.

The AMMI stability showing the relationship between experimental genotypes and test environments for PYLP, HPW, HKW and SP across three seasons is presented in Fig. 5a–d. For PYLP, HPW, HKW and SP, sum22; kh20 and kh21; kh20; and kh21 respectively, showed weak interaction forces. The genotypes, PBS 16028, PBS 16032, PBS 16046, and PBS 16053 for PYLP (Fig. 5a); PBS 16021, PBS 16032, PBS 16039, and PBS 16044 for HPW (Fig. 5b); PBS 16016, PBS 16032, PBS 16038, and PBS 16039 for HKW (Fig. 5c); and PBS 16038, PBS 16032 and Dh 86 for SP (Fig. 5d) were superior performing. Out of these genotypes, PBS16028 for PYLP; PBS 16021 and PBS 16039 for HPW, PBS 16016 and PBS 16032 for HKW, and PBS 16038 and Dh 86 for SP was placed on zero PC1 scoreline, or closer to PC1 line, making it more stable with wider adaptation to the test environments. PBS 16032 though had highest HPW, but was found to be unstable specifically adapted to the test environment. Similarly, PBS 16038 had specific adaptation to the test environment for HKW.

### Comparative performance and stability of genotypes differing in yield and related traits as assessed by ASI

For PYLP, based on ASI, PBS 15044, 16017, 16035, 16038, and 16045 were stable across seasons. While in terms of stability, based on ASI, PBS 16013, 16017, 16020, 16035 and 16039 for HPW; PBS 16025, 16031, 16045, 16046, 16047 for HKW and Girnar 3, Dh 86, PBS 16015, 16031, 16035 for SP were superior (Table 5). Simultaneous selection indices (SSI) for genotypes were calculated using the sum of ASI ranks and genotype ranks determined from pod yield and related traits^9^. The genotypes with the lowest SSI values are the most stable and function well across environments. Based on SSI, PBS 15044, 16025, 16027, 16028, 16029, and 16035 for PYLP; PBS 16020, 16021, 16035, 16038 and 16039 for HPW; 16015, 16016, 16025 and 16053 for HKW; Dh 86, PBS 16015, 16028, 16031, and 16038 for SP were stable with high mean values for studied traits. The ranking of each genotype based on SSI score is presented in Table 5.

**Table 5:** Comparative ranks of AMMI stability index (ASI) and simultaneous selection index (SSI) for yield and related traits in groundnut.

	PYLP			HPW			HKW			SP		
	rASI	rY	SSI	rASI	Ry	SSI	rASI	rY	SSI	rASI	rY	SSI
Dh 86	23	30	53	15	31	46	28	21	49	4	3	7
Girnar 3	13	22	35	14	22	36	32	10	42	3	17	20
PBS 15044	4	10	14	17	4	21	18	5	23	11	13	24
PBS 16004	22	9	31	11	10	21	8	13	21	16	19	35
PBS 16013	18	12	30	3	16	19	15	17	32	22	4	26
PBS 16015	30	17	47	29	13	42	9	9	18	2	12	14
PBS 16016	7	27	34	31	23	54	14	4	18	24	24	48
PBS 16017	2	32	34	4	20	24	7	23	30	19	25	44
PBS 16020	17	13	30	5	7	12	23	11	34	23	9	32
PBS 16021	15	21	36	7	2	9	26	8	34	25	31	56
PBS 16024	16	29	45	32	15	47	25	29	54	30	26	56
PBS 16025	6	5	11	9	11	20	2	12	14	7	18	25
PBS 16026	29	14	43	25	32	57	17	27	44	27	32	59
PBS 16027	20	6	26	12	24	36	13	19	32	8	8	16
PBS 16028	8	1	9	18	18	36	24	30	54	6	6	12
PBS 16029	11	15	26	13	25	38	16	24	40	9	7	16
PBS 16031	25	7	32	21	29	50	4	31	35	5	5	10
PBS 16032	28	2	30	30	1	31	30	1	31	17	2	19
PBS 16035	1	16	17	2	8	10	6	15	21	1	20	21
PBS 16037	19	23	42	24	14	38	22	7	29	18	28	46
PBS 16038	5	28	33	6	5	11	31	3	34	10	1	11
PBS 16039	10	24	34	1	3	4	27	2	29	14	10	24
PBS 16041	9	18	27	16	30	46	19	25	44	26	11	37
PBS 16042	27	20	47	22	27	49	20	22	42	13	27	40
PBS 16044	21	19	40	10	28	38	12	26	38	12	14	26
PBS 16045	3	25	28	8	19	27	3	18	21	28	16	44
PBS 16046	32	3	35	19	6	25	5	14	19	20	23	43
PBS 16047	24	26	50	26	9	35	1	20	21	15	15	30
PBS 16051	12	31	43	27	26	53	29	32	61	32	21	53
PBS 16052	14	11	25	28	17	45	11	28	39	21	29	50
PBS 16053	26	4	30	23	12	35	10	6	16	29	22	51
TPG 41	31	8	39	20	21	41	21	16	37	31	30	61

*PYLP* pod yield/plant, *HPW* hundred pod weight, *HKW* hundred kernel weight, *SP* shelling percentage.

### Identification of stable and productive genotypes by BLUP

**Figure 6:**
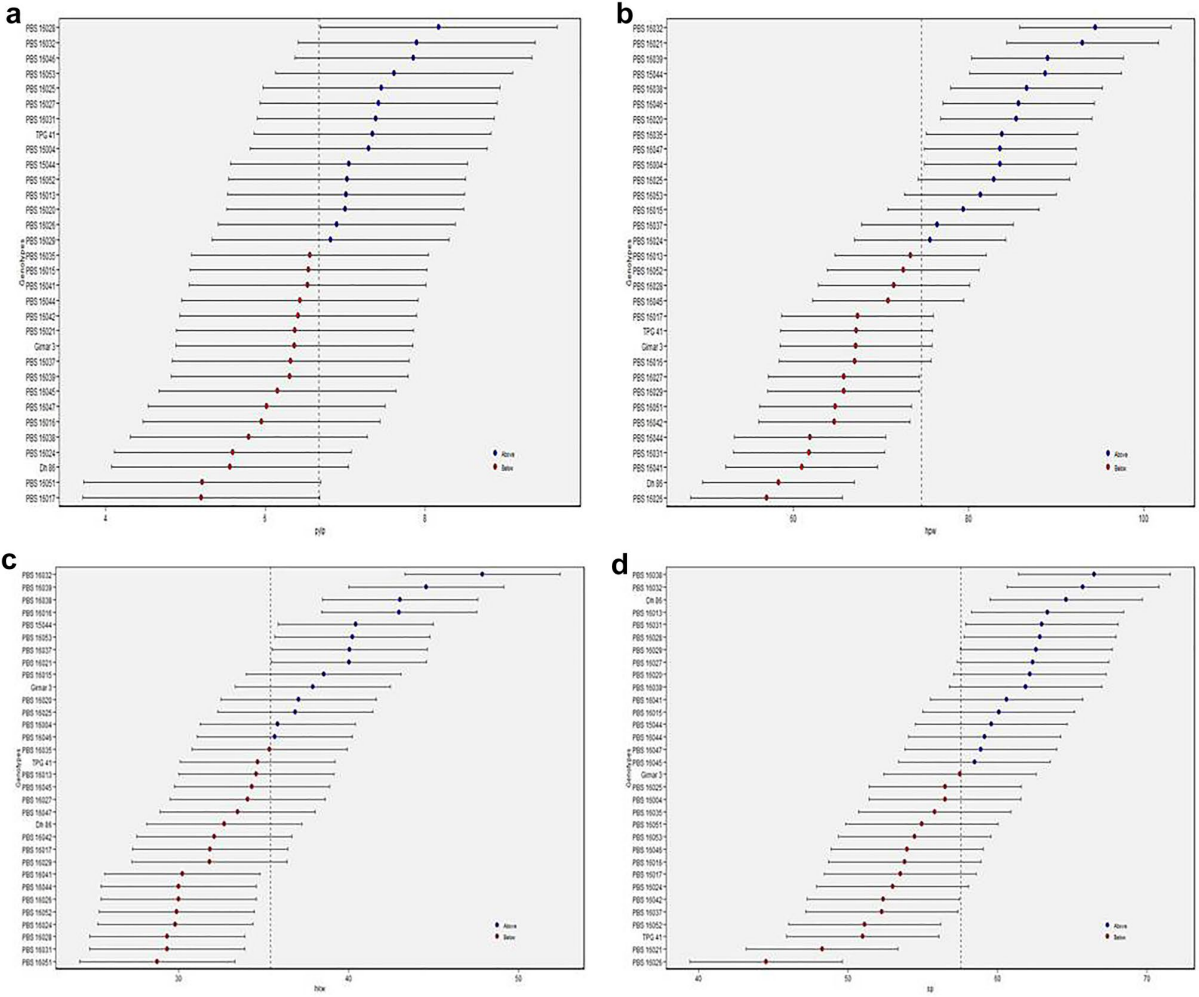
Identification of stable and productive genotypes by BLUP of 32 groundnut genotypes evaluated under three seasons *viz*., kh21, sum22, and kh22 (**a**) PYLP, (**b**) HPW, (**c**) HKW, (**d**) SP.

PBS 16028, 16032, and 16046 for PYLP (Fig. 6a); PBS 16021, 16032, 16039 for HPW (Fig. 6b); PBS 16032, 16039, 16038 for HKW (Fig. 6c); PBS 16038, 16032 and Dh 86 (Fig. 6d) for SP, were top three genotypes with the highest predicted means. Among other genotypes with predicted means above grand means from BLUP estimates were, PBS 16053, 16025, 16027, 16031, TPG 41, 16004, 15044, 16052, 16013, 16020, 16026, and 16029 for PYLP; PBS 15044, 16038, 16046, 16020, 16035, 16047, 15004, 16025, 16053, 16015, 16037, and 16024 for HPW; PBS 16016, 15044, 16053, 16037, 16021, 16015, Girnar 3, 16020, 16025, 16004, 16046 for HKW; PBS 16013, 16031, 16028, 16029, 16027, 16020, 16039, 16041, 16015, 15044, 16044, 16047, and 16045 for SP. So, a similar ranking of genotypes was observed for pod yield using BLUPs as from mean yield data over environments.

### Comparative performance of genotypes as assessed by YREM

Further, in our study the average YREM values for PYLP, HPW, HKW and SP ranged from 0.41 (PBS 16017) to 0.89 (PBS 16028); 0.56 (PBS 16026) to 0.97 (PBS 16032); 0.55 (PBS 16028, 16051) to 0.89 (PBS 16039); 0.62 (PBS 16026) to 0.99 (PBS 16038) that indicate 11 to 59%; 3 to 44%; 11 to 45%; 1 to 38% respective loss of attainable yield due to crossover GSI (Table 6). The comparative stable genotypes based on YREM values were, PBS 16028, 16032, 16053 and 16046 for PYLP; PBS 15044, 16021, 16032, and 16039 for HPW, PBS 16038, 16039, 16016 and 16032 for HKW; and PBS 16013, 16038, 16039, 16020, 16027, 16028, 16029, 16031 and 16032 for SP.

**Table 6:** Estimates of YREM’s of groundnut genotypes for yield and related traits.

	PYLP				HPW					HKW				SP		
	kh20	kh21	sum22	Avg	kh20	kh21	sum22	Avg	kh20	kh21	sum22	Avg	kh20	kh21	sum22	Avg
Dh 86	0.57	0.57	0.30	0.48	0.61	0.61	0.48	0.57	0.82	0.53	0.57	0.64	0.96	0.95	0.97	0.96
Girnar 3	0.68	0.54	0.60	0.60	0.64	0.70	0.64	0.66	0.88	0.48	0.90	0.75	0.82	0.83	0.87	0.84
PBS 15044	0.81	0.75	0.60	0.72	0.92	0.93	0.87	0.91	0.86	0.76	0.80	0.81	0.86	0.85	0.91	0.87
PBS 16004	0.85	0.88	0.55	0.76	0.91	0.85	0.80	0.85	0.79	0.66	0.67	0.70	0.74	0.85	0.87	0.82
PBS 16013	0.70	0.66	0.72	0.69	0.74	0.79	0.67	0.73	0.80	0.65	0.59	0.68	0.86	0.96	1.00	0.94
PBS 16015	0.80	0.80	0.35	0.65	0.88	0.92	0.62	0.81	0.87	0.74	0.69	0.77	0.84	0.91	0.90	0.88
PBS 16016	0.57	0.60	0.43	0.53	0.78	0.75	0.48	0.67	1.00	0.80	0.81	0.87	0.75	0.83	0.75	0.77
PBS 16017	0.40	0.45	0.37	0.41	0.73	0.66	0.62	0.67	0.67	0.61	0.56	0.61	0.73	0.83	0.75	0.77
PBS 16020	0.64	0.69	0.72	0.68	0.93	0.88	0.81	0.87	0.85	0.73	0.63	0.74	0.87	0.90	0.99	0.92
PBS 16021	0.67	0.70	0.45	0.61	0.99	1.00	0.87	0.95	0.90	0.70	0.80	0.80	0.62	0.63	0.78	0.68
PBS 16024	0.39	0.46	0.52	0.46	0.93	0.88	0.51	0.77	0.64	0.60	0.47	0.57	0.62	0.74	0.91	0.75
PBS 16025	0.83	0.77	0.72	0.77	0.89	0.85	0.79	0.84	0.80	0.69	0.68	0.73	0.80	0.81	0.86	0.82
PBS 16026	0.58	0.62	0.78	0.66	0.69	0.54	0.45	0.56	0.65	0.57	0.50	0.57	0.60	0.65	0.59	0.62
PBS 16027	0.89	0.88	0.57	0.78	0.70	0.69	0.57	0.65	0.78	0.62	0.61	0.67	0.91	0.93	0.93	0.92
PBS 16028	0.98	0.88	0.83	0.89	0.77	0.77	0.61	0.72	0.57	0.60	0.49	0.55	0.90	0.93	0.96	0.93
PBS 16029	0.54	0.73	0.67	0.64	0.72	0.68	0.57	0.65	0.63	0.58	0.62	0.61	0.91	0.94	0.93	0.93
PBS 16031	0.65	0.74	0.82	0.74	0.62	0.59	0.61	0.60	0.63	0.54	0.51	0.56	0.89	0.95	0.96	0.93
PBS 16032	1.00	1.00	0.59	0.86	0.94	0.96	1.00	0.97	0.95	0.95	1.00	0.97	0.98	1.00	0.96	0.98
PBS 16035	0.55	0.72	0.56	0.61	0.92	0.86	0.79	0.86	0.75	0.65	0.67	0.69	0.77	0.82	0.83	0.81
PBS 16037	0.51	0.78	0.44	0.57	0.77	0.77	0.77	0.77	0.85	0.82	0.73	0.80	0.70	0.72	0.82	0.75
PBS 16038	0.40	0.62	0.44	0.49	0.91	0.94	0.81	0.88	0.86	1.00	0.72	0.86	1.00	0.98	1.00	0.99
PBS 16039	0.41	0.66	0.60	0.56	1.00	0.91	0.83	0.92	0.88	0.93	0.87	0.89	0.84	0.94	0.95	0.91
PBS 16041	0.49	0.68	0.62	0.60	0.56	0.63	0.58	0.59	0.69	0.56	0.50	0.58	0.80	0.90	0.96	0.89
PBS 16042	0.38	0.61	0.70	0.56	0.80	0.59	0.55	0.65	0.72	0.56	0.59	0.62	0.69	0.76	0.80	0.75
PBS 16044	0.59	0.78	0.44	0.60	0.61	0.62	0.58	0.60	0.64	0.57	0.52	0.57	0.81	0.92	0.87	0.86
PBS 16045	0.44	0.64	0.54	0.54	0.76	0.70	0.67	0.71	0.74	0.66	0.61	0.67	0.77	0.85	0.93	0.85
PBS 16046	0.62	0.76	1.00	0.79	0.90	0.87	0.85	0.87	0.79	0.66	0.66	0.70	0.67	0.82	0.83	0.77
PBS 16047	0.62	0.68	0.35	0.55	0.92	0.79	0.85	0.85	0.71	0.62	0.62	0.65	0.78	0.89	0.90	0.86
PBS 16051	0.44	0.49	0.32	0.41	0.62	0.63	0.66	0.63	0.61	0.59	0.44	0.55	0.90	0.87	0.63	0.80
PBS 16052	0.75	0.83	0.54	0.71	0.69	0.71	0.76	0.72	0.57	0.56	0.57	0.56	0.67	0.70	0.81	0.73
PBS 16053	0.85	0.98	0.57	0.80	0.90	0.89	0.70	0.83	0.85	0.79	0.76	0.80	0.87	0.74	0.77	0.79
TPG 41	0.70	0.63	0.88	0.74	0.71	0.74	0.55	0.67	0.76	0.61	0.66	0.68	0.81	0.79	0.60	0.73

*PYLP* pod yield/plant, *HPW* hundred pod weight, *HKW* hundred kernel weight, *SP* shelling percentage.

### Overall identification of superior genotypes for yield and related traits in the genetic background of fresh seed dormancy

From, the cumulative analysis superior performing genotypes *viz*., PBS 16004, 16015, 16021, 16037 and 16041 (IOD15DAS); PBS 16015, 16016, 16021, and 16041 (IOD21DAS) were stable throughout the screening seasons. PBS 16028, 16032, 16046, and 16053, which were among the highest-performing genotypes for PYLP, also exhibited stability in AMMI1 biplot and YREM analysis. Further, PBS 16021, 16032, and 16039 for HPW; PBS 16016, 16032, 16038, and 16039 for HKW; and 16038 and 16032 for SP were superior in terms of performance and stability. Among these, PBS 16028 (PYLP); PBS 16021, 16039 (HPW); PBS 16016 (HKW), and 16038 (SP) showed superiority from SSI analysis. The predicted means for each of the genotypes mentioned were also greater than the grand means as determined by BLUP analysis. Further, among these genotypes PBS 16021, 15044, 16038, and 16039 identified with high HPW were documented to possess a significant degree of dormancy (greater than 90% for duration of 3 weeks). Subsequently, genotypes of high HKW such as PBS 15044, 16016, PBS 16038, and PBS 16039, and with high SP *viz*., PBS 16013, 16031, and 16038, had fresh seed dormancy for a maximum of 3 weeks.

## Discussion

Groundnut (*Arachis hypogaea* L.) is a food legume that is cultivated on a global scale. It is renowned for its substantial protein and unsaturated oil content. However, one of the most significant constraints impeding its production is the in situ sprouting of Spanish groundnuts. Genotypes with over 90% dormancy and 2–3-weeks duration are more successful in regions where precipitation patterns during crop maturity are unpredictable^3^. A study conducted to assess seed dormancy in a U.S. groundnut mini-core collection revealed notable variability among the accessions in terms of fresh seed germination %^25^. The extent to which a plant goes into dormancy (IOD) is mostly determined by its genes, although environmental conditions are also important^26^. The high percentage sum of squares attributable to the "Genotype" factor in the current investigation indicated that genetic variation among genotypes contributed substantially to the observed differences in the IOD. The examination of the Spanish-type groundnut ABLs for dormancy intensity in the field and laboratory and other yield characteristics across multiple seasons unveiled notable variations in the G × S interaction^27^. The presence of an interaction effect implies that the germination percentage exhibits seasonal variation, potentially attributable to non-genetic variables including temperature, precipitation, humidity, solar radiation, and environmental factors. The development of these advanced breeding lines involved the design of varied crosses including dormant genotypes, namely the advanced line or Virginia type (sub spp. *hypogaea* var. *hypogaea*), as one of the parental lines. The results presented in this study align with the findings reported by Kumar et al. (1991) and Kumar et al. (2017), they also observed notable genetic variability in the degree of dormancy. Nevertheless, the significant impact of environmental cues and the reliance on visual screening for cultivar selection pose challenges in achieving fresh seed dormancy. Hence, the utilization of molecular markers linked to the fresh seed dormancy was employed to authenticate the phenotypic findings^28^. In confirmation with the marker data, for IOD of > 90% for up to 2–3 weeks after sowing, PBS 15044, 16004, 16013, 16015, 16016, 16017, 16020, 16021, 16026, 16031, 16035, 16037, 16038, 16039, 16041, and 16042 were desirable. However, to confirm functionality of the marker and its segregation with marker a very large number of genotypes needs to be genotyped. The AMMI stability model is frequently employed in order to understand the GSI pattern and identify cultivars that exhibit stability across seasons or stable cultivars in target environments^9^. The results of the AMMI ANOVA indicate that genotypes, environments and GSI have a statistically significant impact on the intensity of dormancy and other yield attributes that were examined. While, signal is low dimensional and repeatable, noise is a spurious, high dimensional and non-repeatable component of genotype variation across environments^16^. Most of this noise goes to GSI, as it accounts for most of the data’s degree of freedom. The sum of squares due to GSI_Signal_ is greater than the GSI_Noise_ for due to a greater contribution from the additive main effect of genotype and variable seasons. The acceptance of a genotype that exhibits stability in yield within a diverse environment is widely acknowledged by researchers involved in breeding programmes, as it serves to mitigate the risk of yield reduction caused by unfavourable climatic circumstances^29,30^. In instances when genotype performance exhibits variability in a diverse environment, the process of genotype selection becomes challenging. Consequently, stability models, such as AMMI biplots, are being widely employed to identify stable genotypes with high yields^9,31^. The shrinkage property of BLUP^32^ and its ability to exploit genetic correlation among the performances of the same genotype in different environments^23^ maximizes the correlation of true and predictive genotypic values, which is primary objective of plant breeders. Breeders commonly employ a range of statistical methods, including AMMI, GGE biplot, BLUP, YREM, or a combination thereof, to ascertain promising lines from multi-seasons trials (METs). In the present study, AMMI1 is studied due to its higher predicative accuracy attributable to (i) capture signal rich component of GSI and (ii) adjust mean estimates of genotypes closer to their true values ^16,33^.The estimates of YREM, is an intuitive, and genotypes attendance-independent measure of test genotype’s performance^17^. YREM is a dynamic measure of genotypes’ performance, as it varies with the performance of best genotypes in a given environment and the best genotype also varies with the environment. The breeding value of genotypes is represented by BLUP, which is a preferred method for predicting the overall performance of genotypes. AMMI biplot analysis, along with BLUP, can be employed to uncover yield performance and stability in various seasons. This enables the selection of genotypes with wide or particular adaptation, depending on breeding requirements. Yield by environmental PC1 and yield by WAASB from AMMI, can be can be taken into account for this objective. AMMI model, followed by ASI and SSI stability models, AMMI 1 biplots, and BLUP identified the stable genotypes with one more superior economic trait. PBS 16021, 15044, 16038, and 16039 with > 90% dormancy for up to 3 weeks identified with high HPW; PBS 15044, 16016, PBS 16038 and PBS 16039 up to 3 weeks FSD had high HKW; and PBS 16013, 16031, and 16038 with high SP in the genetic background of dormant lines. It is possible to make use of these identified ABLs in order to develop novel peanut varieties that have specified levels of fresh seed dormancy and high yields/other traits by employing appropriate breeding strategies.

## Conclusion

In order to reduce the amount of in situ germination losses that are caused by unexpected rains during harvest or delayed harvesting, it is essential to have a brief period of seed dormancy, which typically lasts between 2 and 3 weeks, particularly in the Spanish bunch type. The identified accessions, which have a dormancy period of two to three weeks and are superior in one or more economic traits, show promise for the breeding of commercial groundnut cultivars that are resistant to pre-harvest sprouting. This would help to reduce losses that are caused by untimely rainfall. The current work not only brought attention to the significance of seed dormancy, but it also brought attention to the possibility of the stable ABLs that were identified for targeted breeding programmes that aim to improve resistance to pre-harvest sprouting in commercially cultivated varieties.

### Supplementary Information

Supplementary Tables.

## Data Availability

All relevant data that support the findings of this study are within the manuscript and in Supporting Information files."

## Abbreviations

HPW: Hundred pod weight
HKW: Hundred kernel weight
SP: Shelling percentage
PYLP: Pod yield per plant
AMMI: Additive main effect and multiplicative interaction
DAS: Days after sowing
GSI: Genotype × season interaction
AMMI: Additive main effect and multiplicative interaction
PHS: Pre-harvest sprouting
FSD: Fresh seed dormancy
METs: Multi-environment trials
IOD: Intensity of dormancy
ASI: AMMI stability index
BLUP: Best linear unbiased prediction
YREM: Yield relative to the environmental maximum
SSI: Simultaneous selection index

## References

[1] Krapovickas, A. Evolution of the genus *Arachis*. In *Agricultural Genetics-Selected Topics* (Moav, R. Ed.). 135–151 (Wiley, 1973).

[2] Upadhyaya HD, Nigam SN (1999). Inheritance of fresh seed dormancy in peanut. Crop Sci..

[3] Naganagoudar YB, Kenchanagoudar PV, Rathod S, Keerthi CM, Nadaf HL, Channappagoudar BB (2016). Inheritance of fresh seed dormancy in recombinant inbred lines (RIL) developed for mapping population TAG 24 × GPBD 4 in groundnut (*Arachis hypogaea* L.). Legum. Res..

[4] Vishwakarma MK, Pandey MK, Shasidhar Y, Manohar SS, Nagesh P, Janila P, Varshney RK (2016). Identification of two major quantitative trait locus for fresh seed dormancy using the diversity arrays technology and diversity arrays technology-seq based genetic map in Spanish-type peanuts. Plant Breed..

[5] Zhang C, Selvaraj JN, Yang Q, Liu Y (2017). A survey of aflatoxin-producing *Aspergillus* sp. from peanut field soils in four agroecological zones of China. Toxins.

[6] Wang ML, Wang H, Zhao C, Tonnis B, Tallury S, Wang X, Clevenger J, Guo B (2021). Identification of QTLs for seed dormancy in cultivated peanut using a recombinant inbred line mapping population. Plant Mol. Biol. Rep..

[7] Gangurde, S.S., Kumar, R., Pandey, A.K., Burow, M., Laza, H. E., Nayak, S.N., Guo, B., Liao, B., Bhat, R.S., Madhuri, N. & Hemalatha, S. Climate-smart groundnuts for achieving high productivity and improved quality: Current status, challenges, and opportunities. In *Genomic Designing of Climate-Smart Oilseed Crops* (Kole, C. eds.). 133–172 (Springer, 2019).

[8] Gangadhara K, Rani K, Ajay BC, Singh S, Kona P, Mori KK, Kumar N, Bera SK, Praharaj CS (2022). Multi-seasons evaluation of Spanish bunch advanced breeding lines for fresh seed dormancy in groundnut (*Arachis hypogaea* L.). Ann. Agric. Res..

[9] Ajay BC, Aravind J, Abdul-Fiyaz R (2019). ammistability: R package for ranking genotypes based on stability parameters derived from AMMI model. Indian J. Genet..

[10] Kumar R, Janila P, Vishwakarma MK, Khan AW, Manohar SS, Gangurde SS, Variath MT, Shasidhar Y, Pandey MK, Varshney RK (2019). Whole-genome resequencing-based QTL-seq identified candidate genes and molecular markers for fresh seed dormancy in groundnut. Plant Biotechnol. J..

[11] Janila P, Manohar SS, Deshmukh DB, Chaudhari S, Papaiah V, Variath MT (2018). Standard Operating Procedures for Groundnut Breeding and Testing.

[12] Miller OH, Burns EE (1971). Internal color of Spanish peanut hulls as an index of kernel maturity. J. Food Sci..

[13] R Development Core Team. *R: A Language and Environment for Statistical Computing*. (R Foundation for Statistical Computing, 2022).

[14] Levene, H. *Contributions to Probability and Statistics: Essays in Honor of Harold Hotelling* (Olkin, L. *et al*. eds.). 278–292 (Stanford University Press, 1960).

[15] Gauch HG, Zobel RW (1988). Predictive and postdictive success of statistical analyses of yield trials. Theor. Appl. Genet..

[16] Gauch HG (2013). A simple protocol for AMMI analysis of yield trials. Crop Sci..

[17] Yan, W. A study on the methodolgy of cultivar evaluation based on yield trial data with special reference to winter wheat in Ontario. Doctoral Dissertation (University of Guelph, 1999).

[18] Jambhulkar NN, Bose LK, Singh ON (2014). AMMI stability index for stability analysis. Central Rice Res. Inst. (Cuttack, Orissa).

[19] Yan W, Hunt LA (1998). Genotype by environment interaction and crop yield. Plant Breed. Rev..

[20] Ajay, B. C., Aravind, J. & Abdul Fiyaz, R. *ammistability: Additive Main Effects and Multiplicative Interaction Model Stability Parameters*. https://CRAN.R-project.org/package=ammistability (2018).

[21] Chuni L, Ajay BC, Chikani BM, Gor HK (2019). AMMI and GGE biplot analysis to evaluate the phenotypic stability of recombinant inbred lines (RILs) of groundnut under mid-season water stress conditions. Indian J. Genet..

[22] Farshadfar E, Mahmodi N, Yaghotipoor A (2011). AMMI stability value and simultaneous estimation of yield and yield stability in bread wheat (*Triticum aestivum* L.). Aust. J. Crop Sci..

[23] Piepho HP, Möhring J, Melchinger AE, Büchse A (2008). BLUP for phenotypic selection in plant breeding and variety testing. Euphytica.

[24] Ajay BC, Abdul FR, Bera SK, Kumar N, Gangadhar K, Kona P, Rani K, Radhakrishnan T (2022). Higher Order AMMI (HO-AMMI) analysis: A novel stability model to study genotype-location interactions. Indian J. Genet..

[25] Wang ML, Chen CY, Pinnow DL, Barkley NA, Pittman RN, Lamb M, Pederson GA (2012). Seed dormancy variability in the US peanut mini-core collection. Res. J. Seed Sci..

[26] Footitt S, Douterelo-Soler I, Clay H, Finch-Savage WE (2011). Dormancy cycling in Arabidopsis seeds is controlled by seasonally distinct hormone-signaling pathways. Proc. Natl. Acad. Sci..

[27] Kumar N, Ajay BC, Dagla MC, Rathnakumar AL, Radhakrishnan T, Lal C, Samdur MY, Mathur RK, Manivel P (2019). Multi-environment evaluation of Spanish bunch groundnut genotypes for fresh seed dormancy. Indian J. Genet..

[28] Bomireddy D, Sharma V, Senthil R, Reddisekhar M, Shah P, Singh K, Reddy DM, Sudhakar P, Reddy BVB, Pandey MK (2024). Identification of donors for fresh seed dormancy and marker validation in a diverse groundnut mini-core collection. Agronomy..

[29] Oladosu Y, Rafii MY, Abdullah N, Magaji U, Miah G, Hussin G, Ramli A (2017). Genotype × environment interaction and stability analyses of yield and yield components of established and mutant rice genotypes tested in multiple locations in Malaysia. Acta Agric. Scand. Sect. B-Soil Plant Sci..

[30] Gangadhara K, Ajay BC, Kona P, Rani K, Kumar N, Bera SK (2023). Performance of some early-maturing groundnut (*Arachis hypogaea* L.) genotypes and selection of high-yielding genotypes in the potato-fallow system. PLoS ONE.

[31] Kumar N, Ajay B, Rathanakumar A, Radhakrishnan T, Lal C, Samdur M, Mathur R, Manivel P, Chikani B (2017). Genetic variability for fresh seed dormancy in Spanish bunch advanced breeding lines of groundnut (*Arachis hypogaea* L.). J. Oilseeds Res..

[32] Searle SR, Casella G, McCulloch CE (1992). Variance Components.

[33] Gauch HG, Piepho H-P, Annicchiarico P (2008). Statistical analysis of yield trials by AMMI and GGE: Further considerations. Crop Sci..

