# Supplementary material for: A comprehensive phenotypic and genotypic evaluation of Spanish groundnuts from diverse crosses to identify superior and stable donors for fresh seed dormancy
Source: Sci Rep. 2024 Jul 1;14:14988. doi: 10.1038/s41598-024-64681-6 (PMC11217502; doi:10.1038/s41598-024-64681-6)
Supplement: Supplementary file 1 — Supplementary Tables. [file 41598_2024_64681_MOESM1_ESM.docx]

**Table S1 The pedigree information of 29 advanced breeding lines (ABLs) of Groundnut**

| **Genotypes** | **Pedigree** | **Habit Group** |
| --- | --- | --- |
| PBS 15044 | TAG 24 X TMV 2NLM | SB |
| PBS 16004 | GAUG 1 X GG 2 | SB |
| PBS 16013 | Girnar 1 X PBS 190 | SB |
| PBS 16015 | Girnar 1 X PBS 190 | SB |
| PBS 16016 | Girnar 1 X PBS 190 | SB |
| PBS 16017 | Girnar 1 X PBS 190 | SB |
| PBS 16020 | Girnar 1 X NRCG 7323 | SB |
| PBS 16021 | Girnar 1 X NRCG 7323 | SB |
| PBS 16024 | GG 2 X PBS 190 | SB |
| PBS 16025 | M 13 X TAG 24 | SB |
| PBS 16026 | M 13 X TAG 24 | SB |
| PBS 16027 | M 13 X TAG 24 | SB |
| PBS 16028 | M 13 X TAG 24 | SB |
| PBS 16029 | M 13 X TAG 24 | SB |
| PBS 16031 | TAG 24 X M 13 | SB |
| PBS 16032 | TAG 24 X M 13 | SB |
| PBS 16035 | TAG 24 X PBS 191 | SB |
| PBS 16037 | TAG 24 X PBS 191 | SB |
| PBS 16038 | ICGS 11 X M 13 | SB |
| PBS 16039 | ICGS 11 X M 13 | SB |
| PBS 16041 | ICGS 11 X M 13 | SB |
| PBS 16042 | ICGS 11 X M 13 | SB |
| PBS 16044 | TAG 24 X ICGS 11 | SB |
| PBS 16045 | TAG 24 X ICGS 11 | SB |
| PBS 16046 | M 13 X SBXI | SB |
| PBS 16047 | SB XI X ICGS 11 | SB |
| PBS 16051 | GG 2 X SG 99 | SB |
| PBS 16052 | TG 37A X SG 99 | SB |
| PBS 16053 | Girnar 2 X SG 99 | SB |

SB, Spanish bunch

**Table S2 Within - environment analysis and descriptive statistics**

| ENV | MSG (df = 31) | MSB (df = 2) | MSE (df = 62) | CV (%) | | h^2^ | AS | Mean |
| --- | --- | --- | --- | --- | --- | --- | --- | --- |
| IOD 15F | | | | | | | | |
| kh21 | **2005.23** | 53.66 | 20.72 | | 5.61 | 0.99 | 0.99 | 81.09 |
| kh22 | **3639.92** | 75.25 | 32.54 | | 7.54 | 0.99 | 1.00 | 75.63 |
| sum22 | **4150.95** | 122.46 | 33.97 | | 8.05 | 0.99 | 1.00 | 72.36 |
| IOD 21F | | | | | | | | |
| kh21 | **3120.19** | 71.31 | 30.85 | | 7.56 | 0.99 | 1.00 | 73.49 |
| kh22 | **4489.62** | 77.43 | 53.65 | | 10.80 | 0.99 | 0.99 | 67.81 |
| sum22 | **4858.43** | 70.97 | 48.85 | | 10.23 | 0.99 | 0.99 | 68.31 |
| IOD15L | | | | | | | | |
| kh21 | **3269.84** | 222.13 | 46.25 | | 9.20 | 0.99 | 0.99 | 73.95 |
| kh22 | **2755.59** | 27.44 | 60.41 | | 9.97 | 0.98 | 0.99 | 77.92 |
| sum22 | **4219.00** | 312.78 | 82.07 | | 12.97 | 0.98 | 0.99 | 69.86 |
| IOD21L | | | | | | | | |
| kh21 | **4757.64** | 11.84 | 30.01 | | 8.38 | 0.99 | 1.00 | 65.33 |
| kh22 | **4247.27** | 298.21 | 147.84 | | 18.21 | 0.97 | 0.98 | 66.77 |
| sum22 | **5182.07** | 242.13 | 45.71 | | 10.31 | 0.99 | 1.00 | 65.59 |

MSS, mean sum of squares; G, genotypes; B, blocks/replications; E, error; CV, co-efficient of variation; h^2^, heritability (broad sense); AS, accuracy of selection; IOD15F, intensity of dormancy 15 days after sowing (field); IOD21F, intensity of dormancy 21 days after sowing; IOD15L, intensity of dormancy 15 days after sowing (laboratory); IOD21L (intensity of dormancy 21 days after sowing, laboratory)

**Table S3 The average performance of genotypes in terms of IOD15F, IOD15L, IOD21F, IOD21L, PYLP, HPW, HKW and SP at Junagadh**

| **S.No** | **Genotypes** | **IOD15F (%)** | **IOD15L (%)** | **IOD21F (%)** | **IOD21L (%)** | **PYLP (g)** | **HPW (g)** | **HKW (g)** | **SP (%)** |
| --- | --- | --- | --- | --- | --- | --- | --- | --- | --- |
|  | Dh86 | 71.95 | 45.19 | 62.96 | 34.82 | 4.83 | 56.29 | 32.25 | 65.83 |
|  | Girnar 3 | 84.45 | 92.60 | 68.06 | 88.53 | 6.16 | 66.22 | 38.30 | 57.50 |
|  | TPG 41 | 95.23 | 95.18 | 93.19 | 90.00 | 7.78 | 66.29 | 34.52 | 49.87 |
|  | PBS 15044 | 99.07 | 96.66 | 98.06 | 90.75 | 7.29 | 90.52 | 41.22 | 59.93 |
|  | PBS 16004 | 99.72 | 100 | 98.15 | 97.96 | 7.70 | 84.72 | 35.88 | 56.31 |
|  | PBS 16013 | 99.44 | 97.77 | 99.17 | 94.45 | 7.23 | 73.22 | 34.44 | 64.37 |
|  | PBS 16015 | 100 | 100 | 100 | 100 | 6.46 | 80.00 | 39.02 | 60.53 |
|  | PBS 16016 | 99.35 | 100 | 98.98 | 100 | 5.48 | 66.11 | 44.17 | 53.16 |
|  | PBS 16017 | 99.26 | 99.63 | 95.92 | 98.52 | 4.24 | 66.42 | 31.29 | 52.80 |
|  | PBS 16020 | 99.17 | 96.66 | 96.11 | 93.34 | 7.22 | 86.76 | 37.32 | 62.97 |
|  | PBS 16021 | 100 | 100 | 99.44 | 99.63 | 6.17 | 95.29 | 40.77 | 46.67 |
|  | PBS 16024 | 77.22 | 73.33 | 70.00 | 64.08 | 4.89 | 75.73 | 28.93 | 52.20 |
|  | PBS 16025 | 25.65 | 23.70 | 15.37 | 8.52 | 7.96 | 83.92 | 37.08 | 56.34 |
|  | PBS 16026 | 98.33 | 98.89 | 96.11 | 98.52 | 7.04 | 54.77 | 29.14 | 42.29 |
|  | PBS 16027 | 31.30 | 19.90 | 18.70 | 2.96 | 7.91 | 64.68 | 33.84 | 63.22 |
|  | PBS 16028 | 8.24 | 18.52 | 0 | 6.30 | 9.15 | 71.09 | 28.38 | 63.78 |
|  | PBS 16029 | 22.60 | 19.26 | 9.53 | 2.96 | 6.91 | 64.66 | 31.27 | 63.48 |
|  | PBS 16031 | 98.61 | 100 | 97.50 | 99.26 | 7.84 | 60.22 | 28.36 | 63.92 |
|  | PBS 16032 | 38.24 | 28.15 | 15.46 | 2.59 | 8.69 | 96.90 | 49.84 | 67.14 |
|  | PBS 16035 | 96.48 | 98.52 | 93.61 | 95.18 | 6.49 | 84.95 | 35.33 | 55.50 |
|  | PBS 16037 | 99.44 | 100 | 97.40 | 97.40 | 6.09 | 76.67 | 40.79 | 51.35 |
|  | PBS 16038 | 98.89 | 97.78 | 97.78 | 96.66 | 5.22 | 88.09 | 44.21 | 68.04 |
|  | PBS 16039 | 98.20 | 97.78 | 91.99 | 94.07 | 6.07 | 90.78 | 46.01 | 62.64 |
|  | PBS 16041 | 100 | 100 | 100 | 100 | 6.43 | 59.29 | 29.40 | 61.14 |
|  | PBS 16042 | 93.20 | 97.78 | 87.60 | 92.96 | 6.24 | 63.46 | 31.57 | 51.47 |
|  | PBS 16044 | 11.30 | 12.96 | 2.78 | 4.45 | 6.28 | 60.34 | 29.14 | 59.42 |
|  | PBS 16045 | 100 | 98.88 | 99.63 | 95.56 | 5.81 | 70.37 | 34.13 | 58.63 |
|  | PBS 16046 | 85.65 | 62.96 | 71.67 | 50.37 | 8.62 | 87.08 | 35.71 | 53.35 |
|  | PBS 16047 | 39.76 | 32.59 | 27.55 | 18.52 | 5.59 | 84.73 | 33.18 | 59.12 |
|  | PBS 16051 | 46.44 | 45.55 | 30.74 | 25.19 | 4.25 | 63.61 | 27.70 | 54.49 |
|  | PBS 16052 | 38.20 | 41.85 | 20.00 | 17.04 | 7.25 | 72.29 | 29.03 | 50.03 |
|  | PBS 16053 | 88.15 | 72.93 | 80.17 | 48.15 | 8.23 | 82.17 | 41.00 | 53.94 |

IOD15F, intensity of dormancy 15 days after sowing (field); IOD21F, intensity of dormancy 21 days after sowing; IOD15L, intensity of dormancy 15 days after sowing (laboratory); IOD21L (intensity of dormancy 21 days after sowing, laboratory); PYLP, pod yield per plant; HPW, hundred pod weight; HKW, hundred kernel weight; SP, shelling percentage
